# Understanding glycan structure and function through artificial intelligence

**DOI:** 10.1016/j.bbadva.2026.100196

**Published:** 2026-06-19

**Authors:** Lachlan Coff, Ujala Bashir, Daniel Bojar

**Affiliations:** a Department of Chemistry and Molecular Biology, University of Gothenburg, 41390 Gothenburg, Sweden; b Wallenberg Centre for Molecular and Translational Medicine, University of Gothenburg, 41390 Gothenburg, Sweden

**Keywords:** AI, Carbohydrate, Glycomics, Mass spectrometry, Biomarker, Lectin

## Abstract

Glycoinformatics has entered the artificial intelligence era. Stymied by a lack of big data, high sequence complexity, and biosynthetic dependencies, the application of machine learning to glycomics data has largely emerged this decade. In this mini-review, we explore the latest groundbreaking computational approaches applied to glycan sequencing, classifying disease risk from glycan biomarkers, and predicting protein–glycan interactions. We detail advancements in the architectures of these models, concluding that they have matured to the stage of extracting predictive information from glycan sequences. We also discuss their challenges and limitations, and how to fully reap their rewards in the future.

## Highlights

• Glycan structures can be annotated from mass spectra using deep learning.
• Trained models can predict disease states from glycomics data.
• Machine learning can predict protein–glycan interactions.

## Introduction

Once a “hard problem”, DeepMind's AlphaFold [1] can now model the three-dimensional structure of proteins from their amino acid sequences with increasing confidence. This problem was overcome using new types of artificial neural networks (the evoformer in AlphaFold 2, followed by the pairformer in AlphaFold 3) trained on a vast dataset of high-resolution protein models. In a sense, AlphaFold “learns” from these data in an analogous manner to humans using inductive reasoning, but machines are capable of superhuman data processing and computing. Overall, machine learning (ML) has revolutionized bioinformatics, helping to close the gap between high-throughput generation of vast “omics” datasets and the ability to meaningfully interpret them. However, glycoinformatics, the subfield of bioinformatics concerned with computational analyses of carbohydrates, has lagged behind computational advancements in genomics and proteomics, partly because the substantial amount of high-quality training data and processing of biological sequences that were instrumental in AlphaFold’s success have developed more slowly in glycoinformatics, as we will discuss here.

Comprising one or more monosaccharides, carbohydrates or glycans are one of the four classes of biomolecules essential for life [45]. Glycans can be linear or branched and are found, e.g., covalently linked to lipids (glycolipids) and proteins (glycoproteins). Glycoconjugates are synthesized by every organism, and are central to cell–cell interactions, including immune functions and pathogenesis. Downstream from the Central Dogma of Molecular Biology, glycans are difficult to measure as well as interpret, and are thus often neglected in multi-omics studies, but are necessary for a holistic understanding of biological phenomena. Consequently, glycoinformatics has advanced in recent years to address key challenges in glycobiology, such as understanding protein–glycan interactions in host–pathogen interactions or elucidating conserved dysregulation in tumor glycosylation. This mini-review summarizes the recent application of ML to glycomics (both in the analysis of mass spectrometry data and the resulting glycomes themselves) and glycan-binding proteins, as well as highlights future directions needed to usher in the age of data-driven glycobiology. For a broader treatment of glycoinformatics and its history, we refer to comprehensive reviews on that topic [8].

## AI-assisted analyses of mass spectrometry data

As with nucleic acid sequencing in transcriptomics, sequencing glycans is fundamental to glycomics. While nanopore-based glycan sequencing, adapting Third-Generation nucleic acid sequencing technologies with ML-driven analysis, is now being explored as a cost-effective method capable of real-time analyses that directly sequence glycans [52], glycomics measurements usually do not “sequence” the molecule in the classic sense. While nuclear magnetic resonance (NMR) can characterize glycans at atomic resolution, resolving monosaccharide isomers and linkages, liquid chromatography coupled with mass spectrometry (LC–MS) is a higher throughput and more sensitive technique that has emerged as the leading method for sequence characterization of glycans from complex biological samples. Specifically, fragment ions in the product ion spectra (MS^2^) of tandem mass spectrometry (MS/MS) can resolve positional isomers. Accurate annotation of glycomics mass spectra is challenging, with each glycan class posing its own challenges. For example, *N*-glycans have a conserved core but can be complex (e.g., up to tetra-antennary, large structures), complicating resolution of positional isomers, whereas *O*-glycans are released by chemical rather than enzymatic cleavage, which can produce “peeling” byproducts in the spectra. Increased automation of glycomics annotation would facilitate the translation of raw data to new biological insights and glycan biomarker candidates. However, currently, analyzing these data requires expertise and is painstakingly manual, limiting the accessibility and impact of these methods in the life sciences as well as clinical contexts.

Early on, computational aids were developed to search spectral databases of known glycans, but Kumozaki et al. [34] were the first to apply ML to such analyses, using structured support vector machines (SVMs) to optimize scoring functions and retrieve better matches. An SVM is a supervised classifier that learns a decision boundary to separate classes, optimizing for maximum distance between the boundary and data points from each class. Although these decision boundaries are linear, kernels (shortcuts of expressing data similarity with high-dimensional data) can be used to separate data via non-linear boundaries. Following a human-interpretable workflow, Urban et al. [43] introduced a decision-tree based method for identifying diagnostic ions from *O*-glycan MS^2^ spectra. Results from such a model may aid expert annotation, but they do not annotate MS^2^ spectra de novo.

**Fig. 1:**
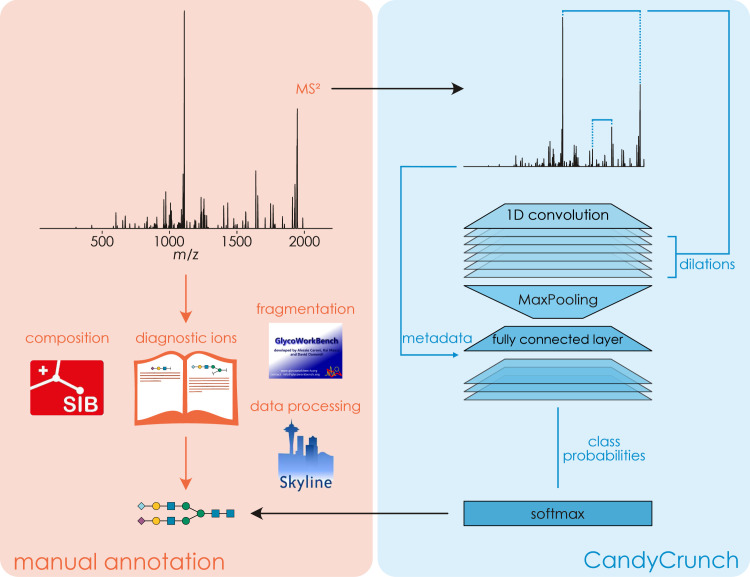
Schematic of manual annotation of a glycomics product ion spectrum (MS^2^), with representative software, in comparison to artificial intelligence de novo annotation, using CandyCrunch as the example. In CandyCrunch, *m*/*z* peaks are binned, and the residual dilated convolutions allow peaks across the spectrum to be considered together. The metadata (glycan class, MS ion mode, ion trap type, LC type and glycan modification type) are embedded in the fully connected layers to yield class probabilities using the softmax function.

Subsequent supervised ML approaches implemented decision trees [14,26] and probabilistic classification [27] to explain glycan annotations from fragmentation spectra. Such classification-based approaches represent a significant advancement in the de novo annotation of MS data. Nevertheless, these tools are limited in their scalability and precision, as they were typically developed and evaluated on very few spectra. To this end, our group released CandyCrunch, a convolutional neural network (CNN) that annotates LC–MS/MS data de novo, in conjunction with our glycowork Python package [42]. The CNN approach has been successful in the hard problem of image recognition, classifying images from a series of connected feature maps that detect local patterns. Analogous local patterns are key to mass spectra annotation, and Kong et al. [32], for instance, applied a CNN in the analyses of glycopeptide spectra. CandyCrunch first groups *m*/*z* peaks from MS^2^ spectra into discrete bins, then learns kernels to construct and pool feature maps in a series of convolutional layers and calculates class probabilities in the final fully connected layer, where dilations allow CandyCrunch to detect patterns across the spectra, reminiscent of ion ratios that are often used by experts in manual annotation (Fig. 1).

A more recent deep learning tool, GlycoBERT (preprint only), implements a transformer architecture initially developed for large language models (LLMs) [2,19]. GlycoBERT first translates peaks and metadata into tokenized sequences or “sentences”, and self-attention of the transformer encoder layers then allows each peak to be weighted in its relationship to each other. The companion model GlycoBART adds glycan sentences and decoder layers, which allow tokens from the MS sentences to be queried against and mapped to tokens from the glycan sentences (i.e., cross-attention). As a generative model, GlycoBART can predict novel structures outside of the training data, which is another step toward the capabilities of expert analyses, although not a replacement. Ultimately, such ML models rely on experimentation to (i) identify diagnostic ions that can explain predictions and (ii) produce curated training sets of annotated spectra that adhere to the Minimum Information Required for A Glycomics Experiment (MIRAGE) [35], especially given the low diversity of the currently largest dataset, exhibiting ∼3400 glycans from ∼500,000 MS^2^ spectra, that has been used to train CandyCrunch and GlycoBERT. We predict that this will have to be combined with approaches that leverage the wealth of unannotated spectra in public data, as shown by CandyCrunch, which was the first such tool to use self-supervised learning on unannotated spectra to expand training data.

Partly because of this, Abtheen et al. [2] suggested further unsupervised training on unannotated datasets and exploration of graph neural networks (GNNs) as a next step in refining automated annotation. Glycans can be more fittingly represented as acyclic molecular graphs (or trees) composed of nodes and edges, a concept recognized in glycoinformatics for decades [3]. Hybrid deep learning approaches incorporating GNNs, such as DeepGP for glycoproteomics, could result in greater performance in analyzing glycomics mass spectra [55]. Burkholz et al. [11] first integrated graph-based glycans with GNNs in SweetNet, followed by Mohapatra et al. [37], and more recent efforts have introduced higher-order GNNs ([30]; M [50]), whereas Kitani et al. [31] used a graph transformer for GlycanGT, using global self-attention between tokenized monosaccharides and linkages within glycans, which exhibited improved predictive performance. As seen in current benchmark studies (M [49]), the complexity of glycan ML models has yet to plateau, and we anticipate further improvements on this front.

## Predicting disease risk using glycan biomarkers

Simply elucidating the glycome of a sample is rarely the end goal of a glycobiology investigation. Instead, the set of glycans is put into a biological context, typically by considering their motifs or sequence features. These features are governed by factors such as glycosyltransferase and glycosidase activities, which can change in response to various stimuli. As such, aberrations in glycosylation have long been recognized as a hallmark of disease, particularly cancer. For example, hypersialylation has been associated with cancer, but a closer examination of glycomics data can reveal specific glycan biomarkers predictive of most diseases. In the past decade, such endeavors have been advanced by more automated AI analyses to identify sensitive as well as specific biomarker candidates. Further, the inclusion of glycomics data in multi-omics studies shows promise for improving the performance of biomarkers or risk scores overall, and ML models trained on such data could be further validated toward their use in clinical diagnostics.

Chocholova et al. [15] were the first to apply ML to the discovery of glycan biomarkers. They characterized the glycoprofiles of serum IgG from clinical samples using lectin assays, and these data were fed into a feedforward neural network (FNN), which used glycan information to improve the classification of samples from patients with rheumatoid arthritis from 83.3% accuracy (clinical markers alone) to 92.5% (clinical markers + lectin-reactivity), although these sample sizes differed by four (84 and 80, respectively).

**Fig. 2:**
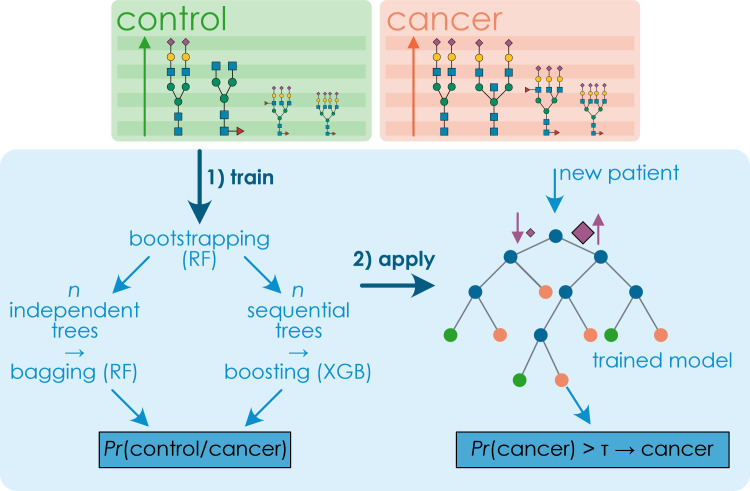
Schematic of decision tree-based machine learning (ML) to classify cancer glycan biomarkers, outlining the basic architecture of both random forest (RF) and Extreme Gradient Boosting (XGB). The relative abundances of glycans from control and cancer datasets are used to train the models. In RF, independent decision trees are constructed from random subsamples (i.e., bootstraps) of the data, considering only a constant number of features with each split. The outputs from the ensemble (i.e., “forest”) of trees are aggregated using a majority rule for the final classification. In XGB, decision trees are trained on the entire dataset, but each sequential tree decreases the loss function of the last (i.e., boosting), so that the ensemble of trees improves the performance of the base learner. The exact classification threshold (τ) is often determined from the ROC curve in a given study.

Since then, many groups have used neural networks or ML algorithms to engage in similar tasks. Demirhan et al. [18] used an FNN to classify tissue samples from 33 patients with gastric cancer and 31 controls using MALDI-MS *N*-glycomics, with an area under the receiver operating characteristic curve (ROC-AUC) of 0.98 from a five-fold cross-validation; their analysis also identified 14 *N*-glycans that significantly differed in mean rank. In classifying serum *N*-glycomes from ovarian cancer patients (*n_control_* = 40 and *n_test_* = 52), Zhang et al. [53] found a random forest (RF) method, which outputs the class predicted by the majority of bootstrapped decision trees, to perform better than an FNN (five-fold ROC-AUC > 0.75 and < 0.56,respectively). In turn, Flevaris et al. [21] found an XGBoost pipeline outperformed RF in a large-scale study of colorectal cancer (*n_control_* = 538 and *n_test_* = 1411), with a five-fold mean ROC-AUC of 0.771 ± 0.025. Extreme Gradient Boosting (XGB) is also a decision tree-based method, but it builds trees sequentially rather than independently from bootstrapped data, each tree correcting errors remaining from the last (Fig. 2). The GlyTrait package uses XGB to classify defined glycan traits or features (e.g., fucosylation, sialylation, galactosylation, etc.), and Fu et al. [23] used this trained model to classify hepatocellular carcinoma (HCC) from serum *N*-glycans (*n_control_* = 110 and *n_test_* = 55), with a mean ROC-AUC (from a nested cross-validation) of 0.915 ± 0.048 across all derived traits. In a larger-scale study, Fu et al. [22] used features derived from GlyTrait in RF models to classify serum *N*-glycomes from 1074 clinical samples as HCC (*n* = 469), cirrhosis (*n* = 218), or chronic hepatitis B (*n* = 223), with mean ROC-AUC values of 0.84 ± 0.07–0.93 ± 0.04.

Other studies used SVM-type models to identify altered glycosylation patterns in HCC. Wang et al. [47] trained SVM models with linear kernels on 83 *N*-glycans from 16 serum glycoproteins to classify HCC samples (*n_control_* = 101 and *n_test_* = 100) with ROC-AUC values of 0.87 ± 0.05–0.91 ± 0.04 in five-fold cross validations, and predictive glycans correlated with spatial omics data from liver tissue. Varghese et al. [44], who included serum *N*-glycomes in a multi-omics study of HCC (*n_control_* = 49 and *n_test_* = 40), found an SVM model to be the top performer of seven ML models. Consistent with Zhang et al. [53], two deep learning models were outperformed by the other classic ML models. Their approach also incorporated recursive feature elimination to train the model using only the most informative features in the final iteration. This SVM-RFE model yielded a five-fold mean ROC-AUC of 0.87 from 29 informative glycans, although no glycans were among the top five features for this model when combined with proteomics and metabolomics data (mean ROC-AUC = 0.844). Like SVMs with non-linear kernels, Quadratic Discriminant Analysis (QDA) employs non-linear decision boundaries to separate classes but constructs these from comparisons between assumed Gaussian distributions. In their comparison of classification models, Vathy-Fogarassy et al. [46] found QDA to outperform an SVM with a Gaussian kernel (mean ROC-AUC = 0.8290 ± 0.0943–0.8410 ± 0.0837 and 0.4456 ± 0.1248–0.7544 ± 0.1109, respectively) in predicting the response of 33 lung cancer patients to chemotherapy from serum *N*-glycan biomarkers characterized by capillary electrophoresis; however, these 33 samples were split into three binary classification tasks (stationary, regression, and progression).

These studies vary considerably in their methodologies, undermining any comparisons. However, they collectively suggest that existing supervised ML algorithms perform well in classifying disease states. Each study uses a fundamentally different approach but can handle the high dimensionality of glycomics data by either using glycan features directly or derived features such as motifs, which could explain their improved performance over linear methods (e.g., logistic regression). In contrast to other applications of ML in glycobiology, researchers have shifted away from modern deep learning models for disease prediction. This is grounded in the well-known observation that classic ML models excel on tabular data [39]. The number of parameters within deep learning models far exceeds the limited number of clinical samples typical of these studies (i.e., overparameterization), which can lead to poor performance on unseen data. Nevertheless, testing multiple ML algorithms for glycan prediction tasks should be considered best practice.

The data collected from samples should also be considered from the perspective of predictive power, as it is not yet known which type of glycomics (*N*-, *O*-, or others) is most predictive on average. Recent multi-omics studies include glycomics, but there remains a dearth of glycoproteomics and *O*-glycomics data that could be paired with *N*-glycomics for enhanced performance [20]. There is also a bias toward serum *N*-glycomics, which follows from ease of specimen collection and sample preparation but risks overlooking more predictive biomarkers in other clinical samples. This points to a general aspect of many current studies that focus on an, in part, narrow subset of the glycome (e.g., IgG glycosylation), which can be very informative and interpretable, yet risks false-negatives and a loss in specificity that might be achieved by richer glycomics measurements. Lastly, biomarkers should also be investigated at the motif-level, as relative changes in these abundances have been shown to exhibit higher statistical power than analyzing full sequences [5], whereas we observe that this is not a currently widespread practice, perhaps due to a lack of structural resolution.

## Predicting protein-glycan interactions

As shown above, glycan motifs (with their link to upstream biosynthetic activity) can be excellent biomarker candidates, yet they are also binding determinants of glycan-binding proteins (GBPs). Enzymes and antibodies can bind to specific glycan substrates or epitopes, whereas lectins (including glycan-binding receptors) have broader selectivities and bind reversibly. Elucidating the binding determinants of GBPs gives greater insight into their biological functions, ranging from host–microbe interactions to immune regulation, as well as their correct use as laboratory reagents, e.g., to stain tissue.

Following the design of nucleic acid and peptide microarrays, glycan microarrays were introduced as a higher throughput method than X-ray crystallography to assess GBP binding specificity. While glycan microarrays can be customized, the Consortium for Functional Glycomics (CFG) and the Glycosciences Laboratory at Imperial College London have each produced standardized glycan microarrays and published the raw data from thousands of experiments [6,24]. This pool of binding data poses a challenge in interpreting the hundreds of data points that are generated with each experiment (e.g., determining motif enrichments in bound vs unbound sequences), let alone the hundreds of thousands in these databases.

Initially, computational tools mined pre-defined motifs or features (e.g., Lewis X) from the binding data. However, undefined features can be extracted from these data using frequent subtree mining, wherein glycans are depicted as graphs or trees and mined for subtrees that are relatively frequent in the binding set but infrequent in the non-binding set. As an early innovation, Hashimoto et al. [25] were the first to apply ML to such a tool, training a soft-margin SVM as its final classifier. Later, CCARL used a logistic regression (LR) classifier to predict the binding of glycans and was shown to outperform the preceding ML tool for 18 out of 20 lectins, with a median ROC-AUC of 0.887 [16]. From an expanded subset of the same CFG dataset, Bojar et al. [9] combined an ensemble decision tree model trained directly on glycan sequences with manual annotation to describe the binding determinants of 57 lectins.

More recently, these efforts have transitioned from classic ML algorithms to deep learning approaches. SweetNet, the successor to the language-based recurrent neural network (RNN) SweetTalk [7], instead learns features by aggregating information within molecular graphs of glycans via a graph convolutional neural network (GCNN), and Burkholz et al. [11] used this model to predict binding determinants of viruses from CFG data. GlyNet, a regression FNN that outputs quantitative predictions, showed improved performance over CCARL for 13 out of 20 lectins, with a mean ROC-AUC of 0.912 [13]. Recently, GlyNet has been further developed into MCNet, which analyzes glycans on the atomic level via an FNN trained on Morgan Fingerprints [12]. Using the same Bidirectional Encoder Representations from Transformers (BERT) architecture as GlycoBERT to construct glycan sentences, GlyBERT (preprint only) then demonstrated more consistent improvements over GlyNet for 18 out of the same 20 lectins, with a median ROC-AUC of 0.960 [17]. The rapid development of these more sophisticated deep learning methods has enabled greater accuracy of predictions that scales with dataset size as well as computational resources and has led to the development of benchmarks to evaluate new model architectures for glycans (M [49]).

**Fig. 3:**
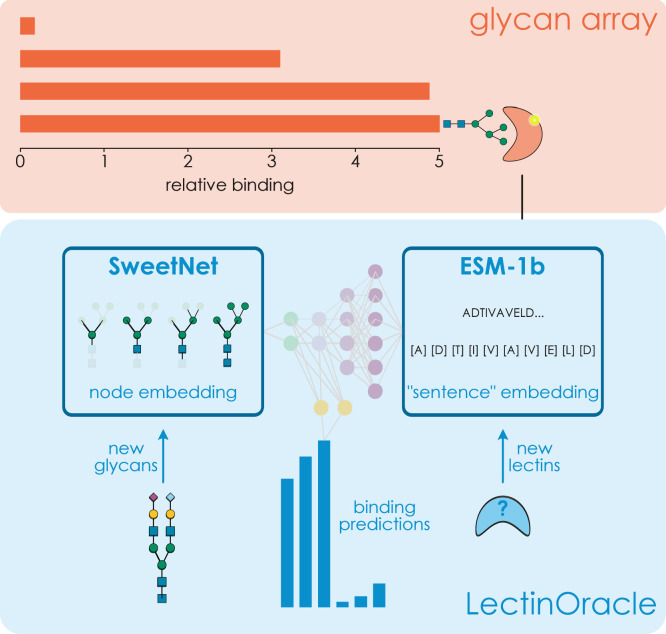
Schematic of predicting protein–glycan interactions from glycan array data, using LectinOracle as the example. Glycan arrary data is used to train LectinOracle, which integrates the graph convolutional neural network of SweetNet for glycan sequences and the transformer of ESM-1b for glycan-binding protein sequences. The trained model can then predict the binding interactions of new glycan and protein sequences.

The sequences of GBPs themselves can also be an input to train such ML models, to circumvent having to train a model for each lectin separately. LectinOracle is a hybrid deep learning tool that incorporates the GCNN of SweetNet for glycan sequences and the transformer-based ESM-1b for protein sequence inputs [36,38] (Fig. 3). LectinOracle was trained on the largest dataset of any preceding tool (over half a million data points) and showed increased performance over GlyNet (90% and 78% accuracy, respectively). It has the unique capability of not only classifying binding glycans from trained GBPs but also zero-shot predictions of binding determinants from new input protein sequences, which can be used to identify new lectins and their binding specificity *in silico*. As a demonstration of this feature, Xu et al. [51] used LectinOracle in the rational discovery of anti-cancer lectins by screening 385 genomes of red algae and cyanobacteria, followed by validating the apoptosis-inducing cytotoxicity of a novel lectin to liver, lung, and colon cancer cell lines in vitro (X [54]).

Molecular representations of glycans are a perpetual challenge in glycoinformatics. Glycans are non-linear polymers with varying linkages between monosaccharides, so do not translate to one-dimensional sequences as readily as nucleic acids and proteins. The molecular graph notation is a closer approximation, and various techniques have extracted additional biological information from them. For example, terminal motifs have been used as a proxy for binding availability, but they are not equivalent in every glycan *ex silico*. The atomic representations of glycans in MCNet and GIFFLAR more closely model the stereochemistry of glycans than monosaccharide-level molecular graphs, and both GIFFLAR and MCNet showed some improved performance over the first version of LectinOracle [12,30]. Predictions of protein–glycan interactions are further improved with consideration of their three-dimensional conformation, which is otherwise flattened by sequence-based and graph-based notations. Thomès et al. [41] showed that when solvent-accessible surface area (SASA), flexibility, ring puckering, polar angle, and torsion angles of glycans from GlycoShape [29] were incorporated into the LectinOracle GCNN (LectinOracle_struct_), the mean squared error (MSE) decreased by over 7% (from 0.598 to 0.557). The approach used by LectinOracle_struct_ could be implemented to predict uncharacterized protein–glycan interactions, but its training is currently limited by the GlycoShape library, which does not include all glycans in the standardized glycan arrays.

Currently, no tool accepts protein structural data as input. It would be interesting to test whether the use of structural data for both glycans and proteins would yield improved performance, especially as GlyBERT, LectinOracle, and MCNet seem comparable in this respect. However, such an approach would increase computational intensity and would be limited by the availability of structural data. Moreover, Lundstrøm et al. [36] showed that LectinOracle was sensitive to substitutions in the binding pocket of a GBP without being trained beyond the sequence-level, and Thomès et al. [41] observed that, while the addition of glycan structural attributes improved the performance of LectinOracle, the trained model largely captures this information prior to its addition, suggesting that structural information can be extracted from glycan sequences, which has been used by Thomès et al. to predict glycan torsion angles directly from sequence. It is possible that the addition of protein structural attributes would have similar diminishing returns.

AlphaFold has revolutionized structural biology with respect to proteins, but its most recent version can also model glycans, whose molecular flexibility poses a challenge in molecular modelling [48]. Huang et al. [28] demonstrated surprising accuracy of predicted oligosaccharide and *N*-glycan models, including for untrained complexes, although glycan modelling is still in its infancy. Likewise, Krishna et al. [33] reported that RoseTTAFold All-Atom accurately predicts branching glycan conformations, even though the model was only trained on glycans comprising up to seven monosaccharides. While predictive modelling tools, such as AlphaFold 3 and RoseTTAFold All-Atom, can bridge the gap between structural biology and downstream molecular docking, there is an underrepresentation of carbohydrates (< 6% of entries) and particularly of polysaccharides in the Protein Data Bank, and molecular docking predictions based on assumptions from AI predictions should be interpreted with caution. Ultimately, while these computational tools can help to guide research, protein–glycan interactions should be verified empirically.

## Conclusion and future directions

Glycoinformatics is the culmination of expertise in chemistry, glycobiology, and computer science, and its impact goes beyond these fields. Here, we reviewed the application of AI to some of the central tasks in glycoinformatics. However, this mini-review is not exhaustive. For example, AI can also be used to identify glycosylation sites, predict the immunogenicity or taxonomic origin of glycans, and elucidate structural dynamics of glycans. Thanks to online repositories, such as GlycoPOST, GlyTouCan, and GlycoShape, glycan-related data is more accessible to researchers than ever before. However, such data must usually first be curated before ML models are trained on them, often in a painstakingly manual process, which is still the bottleneck in both advancing existing tasks as well as opening up new tasks to ML-driven exploration.

While recent years have seen the rise of glycan-specific AI model architectures, many facets of training and applying models on glycan sequences still remain relatively unexplored. Both data augmentation and pre-training, common concepts in general AI, have been largely ignored so far in the case of glycans, which require bespoke methods. CandyCrunch, for instance, has shown that domain-specific loss functions, penalizing the model based on a modified graph distance that accounts for glycan structural ambiguity, can drive further advances in predicting glycan structure and function. This is in line with Bao et al. [5] showing that motifs, due to their direct link to biosynthesis, carry more statistical power than sequences and showcases how the biosynthetic dependencies in glycans can be leveraged via domain knowledge.

Regarding data augmentation, two methods are currently in use for glycans. One, originally proposed and used by SweetTalk, made use of the non-uniqueness of IUPAC-condensed glycan strings and presented the model with various string configurations that all described the same glycan [10]. The other data augmentation strategy, closely mimicking visual perturbations in image-related data augmentation, introduces glycan token wildcarding (e.g., Gal to Hex, or a1–3 to a1-?) to enable the model to learn connections between related tokens. This is for instance used by default in training glycan-related AI models in the glycowork library [40].

Overall, glycoinformatic tools are becoming more complex in their architectures, which has tended toward improved predictions, but aspects such as proper data splitting (e.g., preventing information leakage in the test set) and evaluating models on realistic tasks (e.g., preserving natural class distributions in the test set) are still insufficiently standardized in the community and may lead to the reporting of overoptimistic model performance. To this end, Xu et al. [49] introduced motif fingerprint-based data-splitting for glycans and sequence-based data-splitting for proteins in GlycanML to prevent leakage of biologically related macromolecules in the training and testing set. We note that especially the ML-related usage of glycomics data in the context of multi-omics data is still in its infancy and we anticipate that future advances will be able to leverage more predictive power by approaches such as feature engineering, which could for instance combine information from glycosyltransferase expression and cognate glycan motif features. Moreover, increased computational intensity can create a barrier to entry for researchers with limited computational resources or skills. Wherever possible, glycoinformaticians should thus limit the computational burden of their tools and co-operate toward their inclusion in accessible online platforms, such as projects from the GlySpace Alliance [4].

## Funding

This work was supported by the Swedish Foundation for Strategic Research; the Swedish Research Council; and the University of Gothenburg, Sweden.

## CRediT authorship contribution statement

**Lachlan Coff:** Writing – review & editing, Writing – original draft. **Ujala Bashir:** Writing – review & editing. **Daniel Bojar:** Writing – review & editing, Supervision, Funding acquisition.

## Declaration of competing interest

The authors declare the following financial interests/personal relationships which may be considered as potential competing interests:Daniel Bojar reports financial support was provided by Swedish Research Council. Daniel Bojar reports financial support was provided by Swedish Foundation for Strategic Research. Daniel Bojar reports a relationship with SweetSense Analytics AB that includes: consulting or advisory. D.B. is consulting on glycobiology-related topics via SweetSense Analytics AB. The remaining authors declare no competing interests. If there are other authors, they declare that they have no known competing financial interests or personal relationships that could have appeared to influence the work reported in this paper.

## Data Availability

No data was used for the research described in the article.
